# Synthesis and Aggregation Behavior of Hexameric Quaternary Ammonium Salt Surfactant Tz-6C_12_QC

**DOI:** 10.3390/polym15224396

**Published:** 2023-11-13

**Authors:** Jianjian Jiao, Chi Ma, Linlin Zhang, Fan Li, Tianxu Gao, Lei Wang, Lee Tin Sin

**Affiliations:** 1 China-Spain Joint Laboratory on Material Science, Shenyang University of Chemical Technology, Shenyang Economic and Technological Development Zone, 11th Street, Shenyang 110142, China; 2 School of Health Management, China Medical University, Shenyang North New Area, No. 77 Puhe Road, Shenyang 110122, China; 3 Graduate School of Life Science, Hokkaido University, Kita 10, Nishi 8, Kita-Ku, Sapporo 060-0810, Japan; 4 Department of Chemical Engineering, Lee Kong Chian Faculty of Engineering and Science, Universiti Tunku Abdul Rahman, Jalan Sungai Long, Bandar Sungai Long, Cheras, Kajang 43000, Selangor, Malaysia

**Keywords:** quaternary ammonium surfactant, polysurfactant, aggregation behavior, dissipative particle dynamics

## Abstract

A hexameric quaternary ammonium salt surfactant Tz-6C_12_QC featuring a rigid triazine spacer and six ammonium groups was synthesized. The molecular structure and aggregation behavior of Tz-6C_12_QC were characterized by nuclear magnetic resonance spectroscopy, surface tension, conductivity, dynamic light scattering, and transmission electron microscopy, etc. Dissipative particle dynamics (DPD) simulation was employed to investigate the self-assembly behavior of Tz-6C_12_QC at different concentrations. The rheological behavior of the polyacrylamide/Tz-6C_12_QC system was characterized by shear rheology. The results indicated that Tz-6C_12_QC exhibited superior surface activity and lower surface tension compared to conventional surfactants. Rheology analysis revealed that Tz-6C_12_QC had a significant viscosity reduction effect on polyacrylamide. DLS and TEM indicated that, as the concentration of Tz-6C_12_QC increased, monomer associations, spherical aggregations, vesicles, tubular micelles, and bilayer vesicles were sequentially formed in the solution. This study presents a synthetic approach for polysurfactants with a rigid spacer and sheds light on the self-assembly process of micelles.

## 1. Introduction

Surfactants are a diverse class of chemicals, reaching production volumes of about 20 million tons per year [1]. As the physical and chemical properties of the aqueous phase can be significantly changed by adding a small amount of surfactant, surfactants have been widely used in industry. With the rapid development of the chemical industry, the demand for high-performance surfactants is increasing. Therefore, polysurfactants, which are monomeric surfactants linked by a spacer group, with unique aggregation behavior and higher surface activity than monomer surfactants, have received extensive attention [2,3,4]. In recent years, polysurfactants with unique properties have been demonstrated to be essential for various applications, such as drug delivery [5], nanoparticle synthesis [6,7,8], and the fabrication of polymer surfactant mixing systems [9].

Quaternary ammonium salt surfactants, one of the traditional cationic surfactants, can be synthesized by three primary methods: reacting a higher alkyl halide with a lower tertiary amine, reacting a higher alkyl amine with a lower alkyl halide [10], or reacting a higher alkyl amine with formaldehyde-formic acid [11,12]. Quaternary ammonium salt surfactants are widely utilized in a variety of industrial processes, such as the manufacture of fungicides, fiber softeners, mineral flotation agents, and emulsifiers. In the past decades, numerous quaternary ammonium salt surfactants with novel structures have been synthesized. S. Khan and colleagues [13] synthesized two polyquaternary ammonium salt surfactants with benzene or biphenyl as the spacer groups. They showed that the rigid spacer groups reduced the viscosity and elasticity of the surfactant–polymer system. Carbonate core displacement experiments suggested that the application of this new quaternary ammonium surfactant could be expanded to chemically enhanced oil recovery. Z. Ding and colleagues [14] synthesized a series of novel quaternary ammonium salt surfactants [C_n_H_2n+1_ − O − CH_2_ − CH(OH) − CH_2_-N^+^ − (CH_3_)_2_ − (CH_2_)_2_]_2_Br_2_^−^ (n = 12, 14, 16), which have a short alkyl chain spacer group. They showed that the critical micellar concentration (CMC) of the prepared quaternary ammonium surfactant was one to two orders of magnitude lower than that of the monomeric surfactant. This indicated that polysurfactants exhibited higher surface activity compared to traditional surfactants. However, at present, most of the studies on polysurfactants focus on a low polymerization degree (such as trimer surfactants and tetramer surfactants), and there are few reports on whether surfactants with a high polymerization degree can maintain high surface activity. D. Hu [15] synthesized a series of novel quaternary ammonium salt surfactants, bis-(*N*-(3-alkylamido-propyl)-*N*, *N*-dimethyl)-p-phenylene diammonium dichloride with a rigid spacer group. The study demonstrated that the surface activity decreased with increasing length of the hydrophobic chains and that micellization was a spontaneous, exothermic, and entropy-driven process. Moreover, it was suggested that the increased surface activity of the polysurfactants was not solely influenced by the length of the hydrophobic and hydrophilic head groups. Rather, the spacer length of the polysurfactants also played a significant role in determining their surface activity. These factors had a significant impact on the self-assembly structure and the aggregation morphologies. Unique aggregation morphologies play an important role in the application of polysurfactants. Hence, it is important to study the aggregation behavior of surfactants and the factors influencing this process. Thus, synthesizing a surfactant with a high polymerization degree and incorporating different spacer groups holds immense potential. By varying the spacer groups, researchers can manipulate the intermolecular interactions between surfactant molecules, thereby modulating their aggregation behavior and surface activity. It is important to explore the aggregation behavior of such surfactants as this can provide valuable insights into their self-assembly properties, micellar structures, and stability. It can also contribute to the development of novel surfactants with enhanced performance and applications in various fields.

Conventional experiments to study the self-assembly of surfactants are difficult to perform owing to the extremely short duration of self-assembly. As a result, computer simulations have emerged as a popular alternative in this field because of their high simulation efficiency, low cost, and wide research scope. Dissipative particle dynamics (DPD) simulation is applicable to both large molecular systems and long-duration simulations, rendering it a widely employed method for studying surfactant self-assembly and polymer matrix compatibility [16,17,18,19]. Recently, Y. Wang employed DPD simulation to investigate the self-assembly of hyperbranched macromolecules. Analysis revealed two distinct types of self-assembly behaviors, and the micellar assembly process was explained in detail. P. Wang [20] used DPD to investigate the self-assembly mechanism of surfactants like CTAC, 16-2-16, 16-4-16, and 16-8-16. The study revealed a correlation between the length of the spacer group and micellar behavior. Furthermore, DPD was used by G. Zhou [21] to investigate the aggregation of quaternary ammonium polysurfactant 26-6-26 in three different systems: pure water, a water–ethanol mixture, and a water–oil mixture. DPD simulations provide an essential foundation for studying the aggregation behavior in various solvent systems. In the past decades, DPD simulation has been used to thoroughly study the self-assembly of surfactants by varying the surfactant types [22,23,24,25], solvent systems [26], space groups [27], and concentrations [28], as well as by conducting simulations with hybrid systems [29]. Among them, several studies were conducted to investigate the self-assembly process and self-assembly morphologies of polysurfactants at different surfactant concentrations or degrees of oligomerization. Among these studies, simulations for investigating the effect of rigid segments on the aggregation behavior of polysurfactants have rarely been reported.

In this study, we synthesized a hexameric triazine quaternary ammonium chloride surfactant Tz-6C_12_QC, which features six ammonium groups and a rigid triazine spacer group. Analytical methods, such as Fourier-transform infrared (FT-IR) spectroscopy, nuclear magnetic resonance (NMR) spectroscopy, surface tension and conductivity measurements, and rheological experiments, were used to further understand the chemical structure and surface activity of Tz-6C_12_QC. Dynamic light scattering (DLS) and transmission electron microscopy (TEM) were utilized to determine the size and morphology of Tz-6C_12_QC micelles in an aqueous solution. DPD simulation was used to understand the self-assembly process of Tz-6C_12_QC. Additionally, rheological analysis of the PHIII/surfactant was used to determine the effects of viscosity-reduction. The findings of this study will contribute significantly to the synthesis of high-performance surfactants and current research on surfactant aggregation.

## 2. Experimental

### 2.1. Materials Preparation

Melamine (C_3_H_6_N_6_, 99%), dimethyl sulfoxide (DMSO, C_2_H_6_SO, AR), *N,N*-dimethyl dodecyl tertiary amine (C_14_H_31_N, 98%), nonionic polyacrylamide(PHIII, molecular weight 10 million), dodecyltrimethylammonium chloride (DTAC, C_15_H_34_ClN, 99%), and sodium dodecyl sulfate (SDS, C_12_H_25_O_4_NaS, 97%) were purchased from Macklin Biochemical Technology Co., Ltd. (Shanghai, China). Epichlorohydrin (C_3_H_5_ClO, AR) was purchased from Aladdin Biochemical Technology Co., Ltd. (Shanghai, China). Triethylamine ((C_2_H_5_)_3_N, AR) was purchased from Hengxing Chemical Reagent Co., Ltd. (Tianjin, China). *n*-Hexane (C_6_H_14_, AR) and absolute ethanol (C_2_H_6_O, AR) were purchased from Sinopharm Chemical Reagent Co., Ltd. (Beijing, China).

### 2.2. Synthesis of Tz-6C_12_QC

**Scheme 1:**
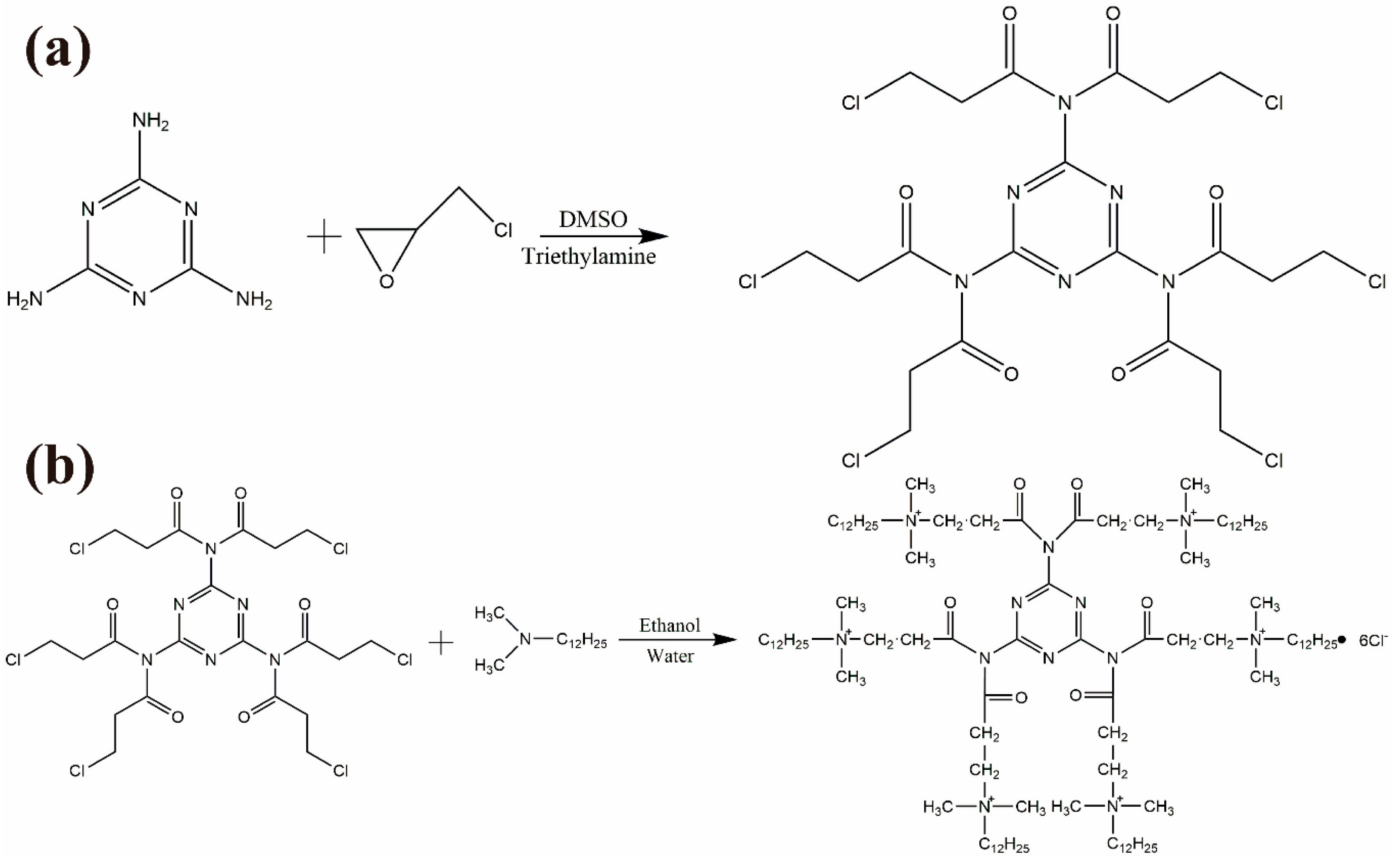
Synthetic scheme of Tz-6C_12_QC, (**a**) intermediate (**b**) Tz-6C_12_QC.

Triethylamine and melamine were dissolved in dimethyl sulfoxide in a three-necked flask at room temperature under a nitrogen atmosphere. Epichlorohydrin with a molar ratio of 9.6:1 to melamine was added slowly to the flask. The mixture was heated to 70–75 °C and allowed to react for 24 h until the solution turned reddish-brown. Subsequently, the solution was concentrated using a rotary vacuum evaporator and purified in a freeze dryer. Finally, the intermediate of Tz-6C_12_QC was obtained. A schematic of the synthesis of the intermediate is shown in Scheme 1a.

The intermediate was dissolved in a three-necked flask containing distilled water, dimethyl sulfoxide, and hydrochloric acid. *N, N*-dimethyl dodecyl tertiary amine dissolved with a molar ratio of 9.6:1 to the intermediate in ethanol was added to the mixture with vigorous stirring. The mixture was refluxed for 16 h at 75 °C. The solvent was removed using a rotary evaporator. The residual solid was washed twice with deionized water, and the water was removed using a rotary evaporator. Finally, the residual solid was dissolved in 100 mL deionized water, which was removed by freeze-drying. A synthetic scheme of Tz-6C_12_QC is shown in Scheme 1b.

### 2.3. Characterizations

#### 2.3.1. FT-IR Analysis

FT-IR spectra of the Tz-6C_12_QC surfactant and its intermediates were recorded on a Nicolet 10 infrared spectrometer (Thermo Fisher Scientific Co., Ltd., Waltham, MA, USA) in the wavelength range of 400–4000 cm^−1^; 16 scans were recorded in each run.

#### 2.3.2. NMR Analysis

NMR analysis of the Tz-6C_12_QC surfactant and the intermediate of Tz-6C_12_QC was performed on an AVANCE III 500 MHz NMR spectrometer (Bruker Analytical Instruments Co., Ltd., Karlsruhe, Germany).

#### 2.3.3. Surface Tension Analysis

Surface tension measurement of the aqueous Tz-6C_12_QC solution was performed on a JK99C Automatic Surface Tension Instrument (Zhongchen Digital Technology Equipment Co., Ltd., Shanghai, China). The Wilhelmy hanging plate method was used to determine the relationship between the surface tension (*γ*) and the concentration (*C*) of the aqueous Tz-6C_12_QC solution. The sampling period was 150 s, and the system temperature was controlled at 25 ± 0.5 °C. The surface tension concentration curve is presented as the empirical Szyszkowski equation for the average value, with error bars corresponding to the standard deviation of three independent experiments [11,30,31].

The empirical Szyszkowski equation is shown in Equation (1):

(1) $$\gamma =\gamma_{0}\left\{1-Blog\left[\left(C/A\right)+1\right]\right\}$$

where $\gamma_{0}$ is the surface tension of the pure solvent, *C* is the surfactant concentration, and *A*, *B* are two empirical adjustable parameters.

The surface excess concentration (*Γ*; mol/m^2^) and the area occupied by each surfactant (*A*; cm^−1^) can be determined from the Gibbs adsorption isotherm equations (Equations (2) and (3)):

(2) $$\Gamma =-\left(d\gamma /d\left(lnC\right)\right)/\left(iRT\right)$$

(3) A=1/NΓ

where *γ* and *C* represent the surface tension and the concentration of the surfactant, respectively. *R* is the gas constant (8.31 J∙K^−1^∙mol^−1^), *N* is the Avogadro constant (6.02 × 10^23^), *T* is the absolute temperature, and the value of *i* (the number of species at the interface whose concentration varies with surfactant concentration) is considered to be 7.

#### 2.3.4. Electrical Conductivity Analysis

The electrical conductivity was analyzed on a DDS-11A Instrument (INESA Scientific Instrument Co., Ltd., Shanghai, China) for establishing the relationship between the electrical conductivity (κ) and the concentration (C) of the aqueous solution of Tz-6C_12_QC. An identical DJS-1D platinum plate with an electrode constant of 0.971 was used for this measurement. The sampling period was 150 s, and the system temperature was controlled at 25 ± 0.5 °C. κ was recorded once there were no fluctuations in the conductivity meter indicator. Every experiment was conducted three times at a particular Tz-6C_12_QC concentration. The conductivity curve is presented as the average value, with error bars corresponding to the standard deviation of three (n = 3) independent experiments.

#### 2.3.5. Rheology Analysis

The rheological properties of the aqueous solution of Tz-6C_12_QC and the solution of PHIII/surfactants, for which the relative mass fraction of PHIII was 1 wt% and the concentrations of the surfactants were 0.10 mmol/L, 0.25 mmol/L, 0.50 mmol/L, and 1.0 mmol/L, were measured using a Discovery HR-2 rheometer (TA waters technology Co., Ltd., Newcastle, DE, USA). The relative mass fraction of PHIII was 1 wt%. The test temperature was controlled at 25 ± 0.5 °C, and steady shear viscosity measurements were conducted over shear rates ranging from 0.01 to 100 s^−1^. The zero-shear viscosity of the solutions was determined by fitting the Carreau–Yasuda model; its model-fitting formula is shown in Equation (4) [32,33,34].

(4) $$\eta -\eta_{\infty }/\eta_{0}-\eta_{\infty }={\left[1+{\left(k\dot{\gamma }\right)}^{a}\right]}^{\left(n-1\right)/a}$$

where $\eta_{0}$, $\eta_{\infty }$ and k represent the zero-shear viscosity, the infinite viscosity, and the consistency, respectively. *n* represents the power law index and a is a parameter describing the transition between the Newtonian plateau and the power law region.

#### 2.3.6. DLS Analysis

DLS experiments were performed on a Zetasizer laser particle analyzer (Malvern Instrument Co., Ltd., Malvern, England) at 25 ± 0.1 °C. Each sample was examined three times, and 16 scans were recorded for each run. The polydispersity and average hydrodynamic diameter (z-average) of the samples were determined from a cumulant analysis of the autocorrelation function.

#### 2.3.7. TEM Analysis

Micrographs of the Tz-6C_12_QC aggregates were obtained using the JEOL JSM-IT800 TEM instrument (Japan Electronics Corporation, Tokyo, Japan) at an operating voltage of 200 kV. To prepare the sample, a drop (2 μL) of the Tz-6C_12_QC solution was placed onto a carbon-coated copper grid (200 mesh) and allowed to stand for 5 min to form a thin liquid film. The sample was then dried before imaging.

### 2.4. Simulation Methods and Models

#### 2.4.1. DPD Simulation Method

In DPD simulations, a certain structure fragment of a single molecule or of several atoms or polymers can be divided into beads using the principle of equal volume. These coarse-grained beads follow Newton’s law [35]. Adjacent beads are considered to interact with each other through the conservative force $F_{ij}^{C}$, the dissipative force $F_{ij}^{D}$, and the random force $F_{ij}^{R}$.

(5) $$dr_{i}/dt=v_{i}$$

(6) $$m_{i}dv_{i}/dt=\sum_{i\neq j}\left(F_{ij}^{C}+F_{ij}^{D}+F_{ij}^{R}\right)$$

where $r_{i}$, $v_{i}$, and $m_{i}$ represent the position vector, the velocity, and the mass of beads, respectively. When $m_{i}dv_{i}/dt=F_{i}$, Equation (7) can be written as:

(7) $$F_{i}=\sum_{i\neq j}\left(F_{ij}^{C}+F_{ij}^{D}+F_{ij}^{R}\right)$$

The beads can interact with each other within a cutoff radius ($r_{c}$). Here, the cutoff radius $r_{c}\equiv 1$. $F_{ij}^{C}$ can be expressed by the following equation:

(8) $$F_{ij}^{C}=f\left(x\right)=\left\{\begin{array}{cc} \alpha_{ij}\left(1-r_{ij}\right)e_{ij}, & r_{ij}<1\\ 0, & r_{ij}>1 \end{array}\right.$$

where $r_{ij}=r_{i}-r_{j}$, $r_{ij}=\left|r_{ij}\right|$, and $a_{ij}$ is a repulsive parameter between beads *i* and *j*. $a_{ij}$ is calculated using the Flory–Huggins parameter. $e_{ij}$ represents a unit vector that points to bead *j* from bead *i*. $F_{ij}^{D}$ and $F_{ij}^{R}$ can be expressed by the following equations:

(9) $$F_{ij}^{D}=f\left(x\right)=\left\{\begin{array}{cc} -\gamma \omega^{D}\left(r_{ij}\right)\left(e_{ij}\cdot v_{ij}\right)e_{ij}, & r_{ij}<1\\ 0, & r_{ij}>1 \end{array}\right.$$

(10) $$F_{ij}^{R}=f\left(x\right)=\left\{\begin{array}{cc} \sigma \omega^{R}\left(r_{ij}\right)\theta_{ij}e_{ij}, & r_{ij}<1\\ 0, & r_{ij}>1 \end{array}\right.$$

where $v_{ij}=v_{i}-v_{j}$, and $\omega^{D}$ and $\omega^{R}$ are the weight functions associated with *r*. Only one of the two weighting functions can be selected arbitrarily while the other one is determined uniquely [35]. These parameters can be represented by the following equations:

(11) $$\omega^{D}\left(r\right)={\left[\omega^{R}\left(r\right)\right]}^{2}$$

(12) $$\sigma^{2}=2\gamma k_{B}T$$

where $k_{B}$ is the Boltzmann constant and *T* represents the temperature. The interaction force between adjacent particles is expressed by the harmonic force,

(13) $$F_{ij}^{bond}=-k_{s}\left(r_{ij}-r_{0}\right)e_{ij},$$

where $k_{s}$ is the elastic constant, and $r_{0}$ represents the equilibrium distance between beads *i* and *j*.

#### 2.4.2. Model and Interaction Parameters

**Figure 1:**
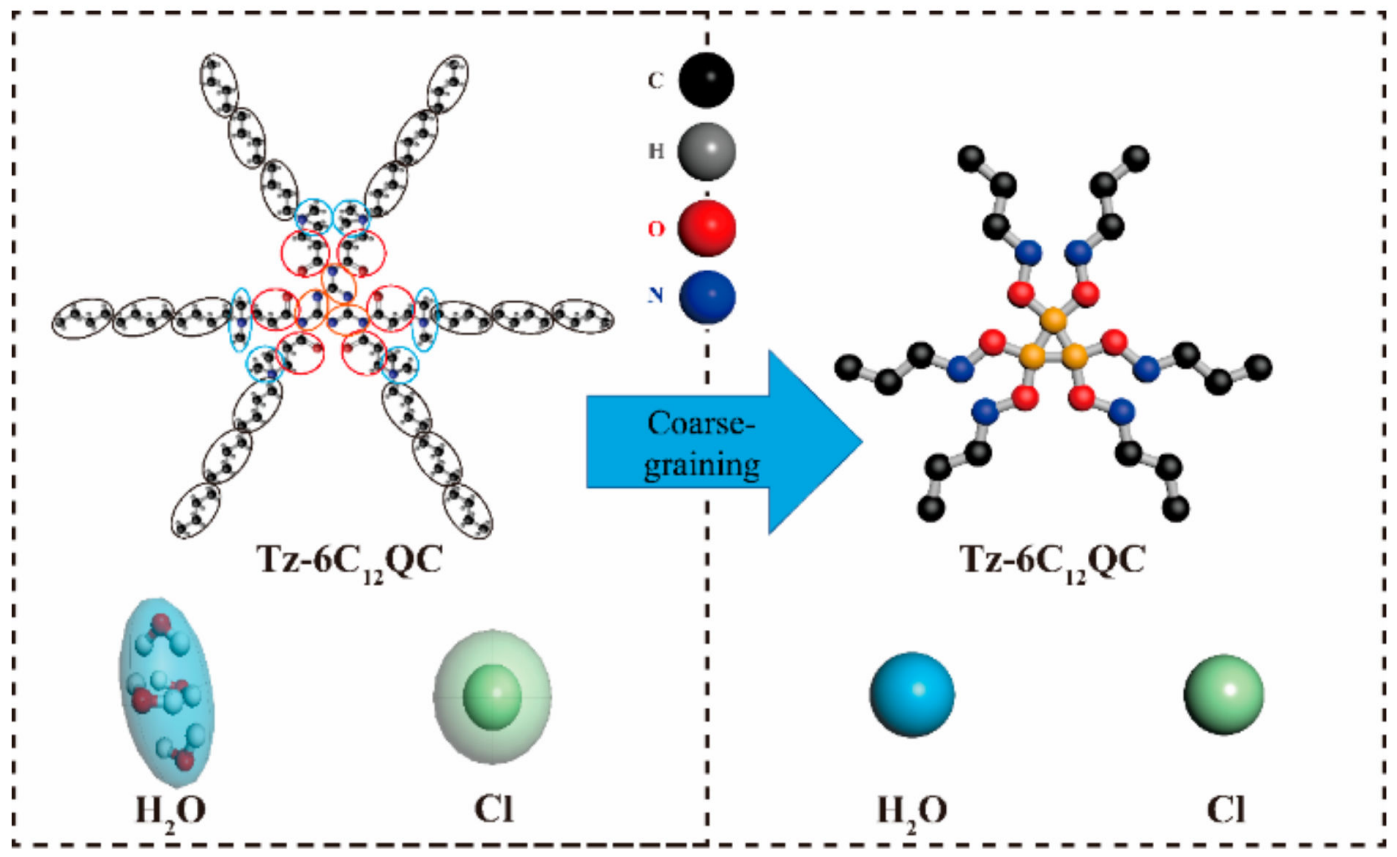
Coarse-grained structural model of Tz-6C_12_QC, H_2_O, and Cl^−^.

The simulation system included Tz-6C_12_QC and water. In the DPD simulation, the surfactant Tz-6C_12_QC was divided into six kinds of beads, namely, A, B, C, D, E, and F, with each bead representing specific chemical components. The coarse-grained structures are shown in Figure 1. The A beads represent -CN_2_, which is the basic structural unit of the triazine spacer group, and three A beads represent a triazine spacer group. Beads B, C, D, E, and F represent oxyhydrophilic groups, quaternary ammonium groups, -C_4_H_8_ hydrophobic groups, -C_4_H_9_ hydrophobic groups, and chloridion, respectively. One G bead consists of four water molecules. In DPD simulations, the most important parameter is the repulsive parameter *a*, which reflects the interaction among the beads. The repulsive force between different beads is determined by the repulsive parameter. Groot and Warren associated the Flory–Huggins parameters with the repulsive parameters and transformed them into solving the Flory–Huggins parameters [36].

The repulsive parameters for the same beads are calculated according to the following equation:

(14) $$a_{ii}=\left(16N_{m}-1\right)k_{B}T/0.2\rho$$

where ρ = 3 and $k_{B}T$ = 1 [21]. *N_m_* represents the number of water molecules. Thus, *a_ii_* is calculated to be 105 [37].

The interaction parameters for different beads can be solved using the following equation [38,39]:

(15) $$a_{ij}=a_{ii}+\chi_{ij}/0.231$$

The Flory–Huggins parameters can be obtained according to the following equations:

(16) $$\chi_{ij}=z\left[E_{ij}-(E_{ii}+E_{jj})/2\right]/RT$$

(17) $${\langle E_{ij}\rangle }_{T}=\int EP_{ij}\left(E\right)e^{-\frac{E}{RT}}dE/\int P_{ij}\left(E\right)e^{-\frac{E}{RT}}dE$$

where *z* represents the coordination number, $E_{ij}$ represents the mixing energy between beads *i* and *j*, *R* is the ideal gas constant, and *T* is the absolute temperature. The $\chi_{ij}$ values were obtained from the Materials Studio 8.0 software using the Blends module. The repulsive parameters are listed in Table 1.

The dimensions of the simulated box are as follows: *L_x_* = 100 Å, *L_y_* = 100 Å, and *L_z_* = 100 Å. The bead density of this system is 3 g/cm^3^, the time step is 100 fs, and the simulation time is 100 ns.

## 3. Results and Discussion

### 3.1. Structure of Tz-6C_12_QC and Its Intermediate

**Figure 2:**
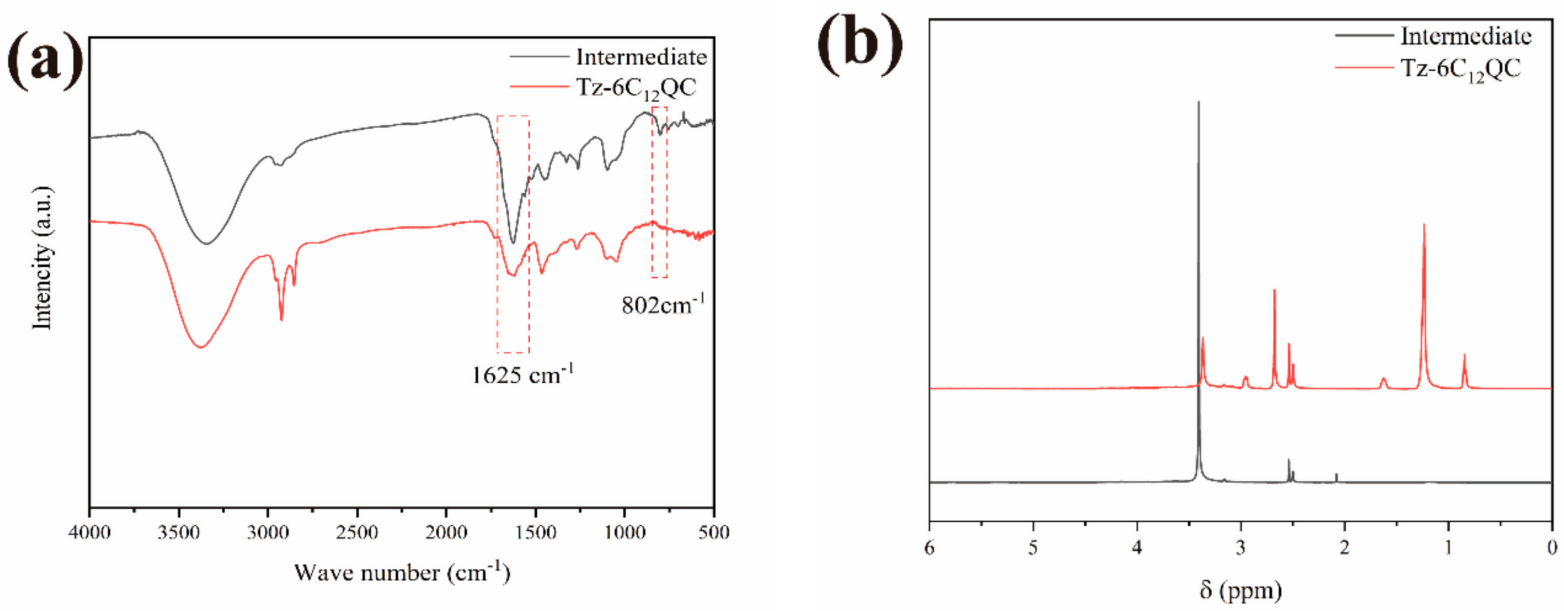
FT-IR (**a**) and ^1^H-NMR (**b**) spectra of Tz-6C_12_QC and its intermediate.

The chemical structure of Tz-6C_12_QC was characterized by FT-IR and ^1^H-NMR spectroscopy. The peak at 1625 cm^−1^ in the two FT-IR spectra (Figure 2a) corresponds to the amide stretching vibration, indicating the presence of amide groups in both the intermediate and Tz-6C_12_QC. Furthermore, the intermediate spectrum showed a peak at 802 cm^−1^, corresponding to the stretching vibration of - CH_2_Cl. The appearance of this characteristic peak suggested that the epichlorohydrin epoxy groups successfully reacted with the melamine amino groups to form the intermediate compound. The disappearance of the peak at 805 cm^−1^ in the Tz-6C_12_QC spectrum implied that the *N, N*-dimethyl dodecyl tertiary amine and the intermediate reacted completely. The ^1^H-NMR spectrum of the intermediate (Figure 2b) showed peaks at δ2.49 (s, 6D, DMSO-d6), δ3.41 (s, 12H, –CH_2_Cl), and δ2.08 (s, 12H, –COCH_2_), indicating the successful formation of the intermediate. The ^1^H-NMR spectrum of Tz-6C_12_QC showed peaks at δ2.49 (s, 6D, DMSO-d6), δ0.83 (s, 18H, -CH_3_), δ1.22 (s, 60H, –(CH_2_)_10_), δ1.61 (s, 12H, –COCH_2_), δ2.66 (s, 12H, –N^+^CH_2_), δ2.94 (s, 36H, –N^+^(CH_3_)_2_), and δ3.35 (s, 12H, –CH_2_CH_2_N^+^), confirming the successful synthesis of Tz-6C_12_QC.

### 3.2. Surface Activity of Tz-6C_12_QC

The surface tension is defined as the energy required to create a unit area of an interphase [40]. Surfactants play a crucial role in lowering the surface tension of water. When micromolar amounts of a surfactant are added to water, the water–air interface is occupied by surfactant molecules, with the hydrophilic headgroup pointing toward the water and the hydrophobic chain pointing toward the air. The surface tension decreases because of the increased surfactant packing at the surface. When the surfactant molecules reach the condition of maximum packing, they begin to aggregate in the bulk solution, forming spheroidal aggregates known as micelles [41]. The CMC is usually identified as the inflection point in the surface tension versus concentration curve. The CMC can also be determined from conductivity or turbidity measurements or from specific fluorescence signals. In this study, the CMC of Tz-6C_12_QC was determined by surface tension, conductivity, and rheological measurements.

**Figure 3:**
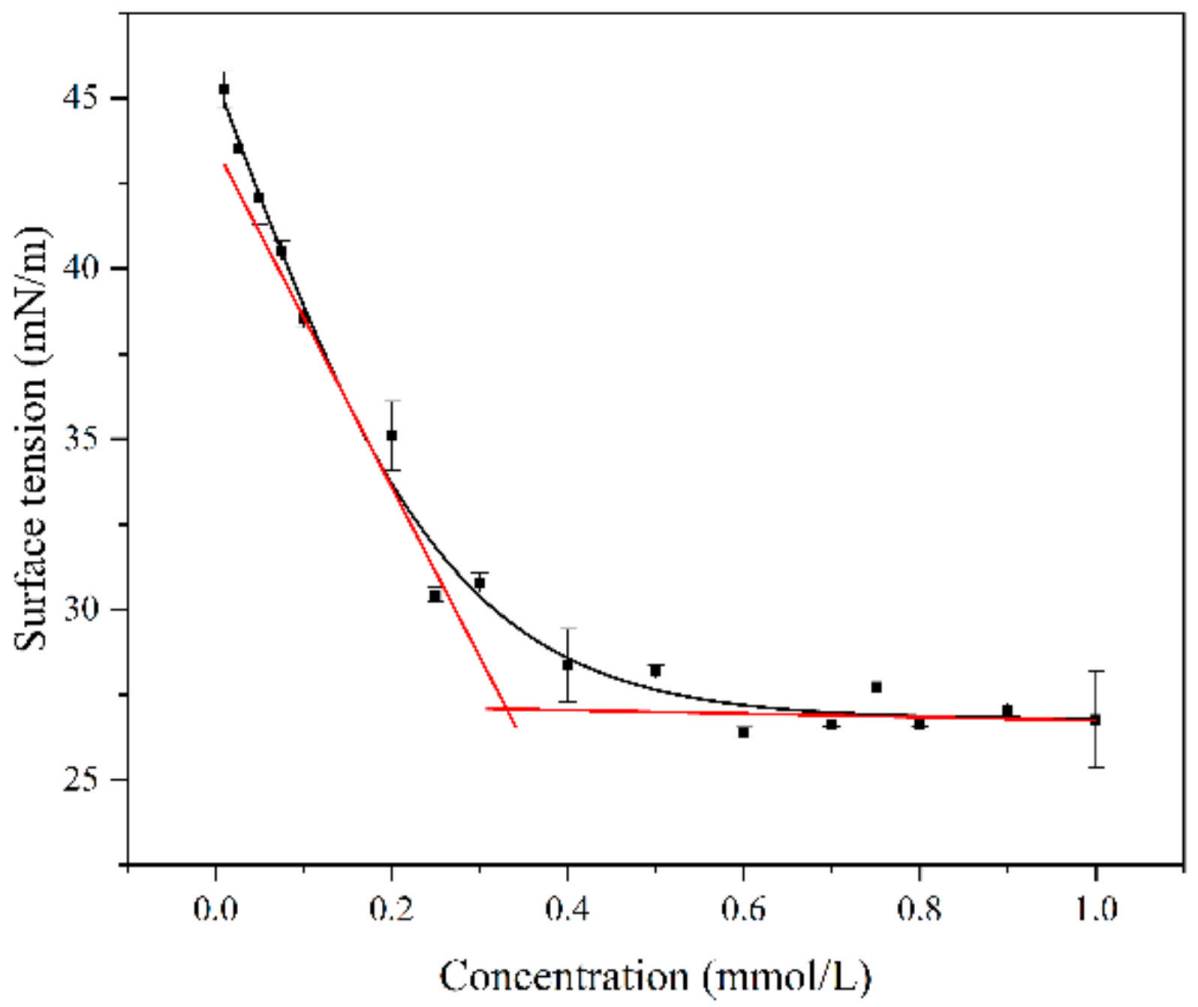
Variation in the surface tension of the aqueous Tz-6C_12_QC solution with concentration at 25 °C, (Black is the surface tension fitting curve, and red is the curve tangent).

As shown in Figure 3, the surface tension of the system significantly decreased with increasing Tz-6C_12_QC concentration. However, it is worth noting that the rate of decrease in the surface tension was not uniform throughout the range of the Tz-6C_12_QC concentrations investigated. The CMC, determined from the inflection point, was 0.34 mmol/L. Compared with dodecyltrimethylammonium bromide (DTAB), which has the same length of alkyl chain and a CMC of 14 mmol/L [42], the hexameric surfactant Tz-6C_12_QC exhibited superior surface activity and a greater propensity for micelle formation. This was because the triazine spacer group increased the regional charge density of the hydrophilic head group and weakened the repulsion between the cationic groups, thereby facilitating the micellization of Tz-6C_12_QC and lowering its CMC. Moreover, as shown in Figure 3, the surface tension of Tz-6C_12_QC (γ_max_ = 28.27 mN/m) was lower than that of DTAB (γ_max_ = 38.6 mN/m). This can be attributed to the presence of the triazine spacer group, which reduced the surface adsorption volume of Tz-6C_12_QC. Consequently, Tz-6C_12_QC had a higher adsorption capacity at the water/air interface, which substantially lowered the surface tension. Overall, Tz-6C_12_QC was more effective in lowering the surface tension than DTAB.

**Figure 4:**
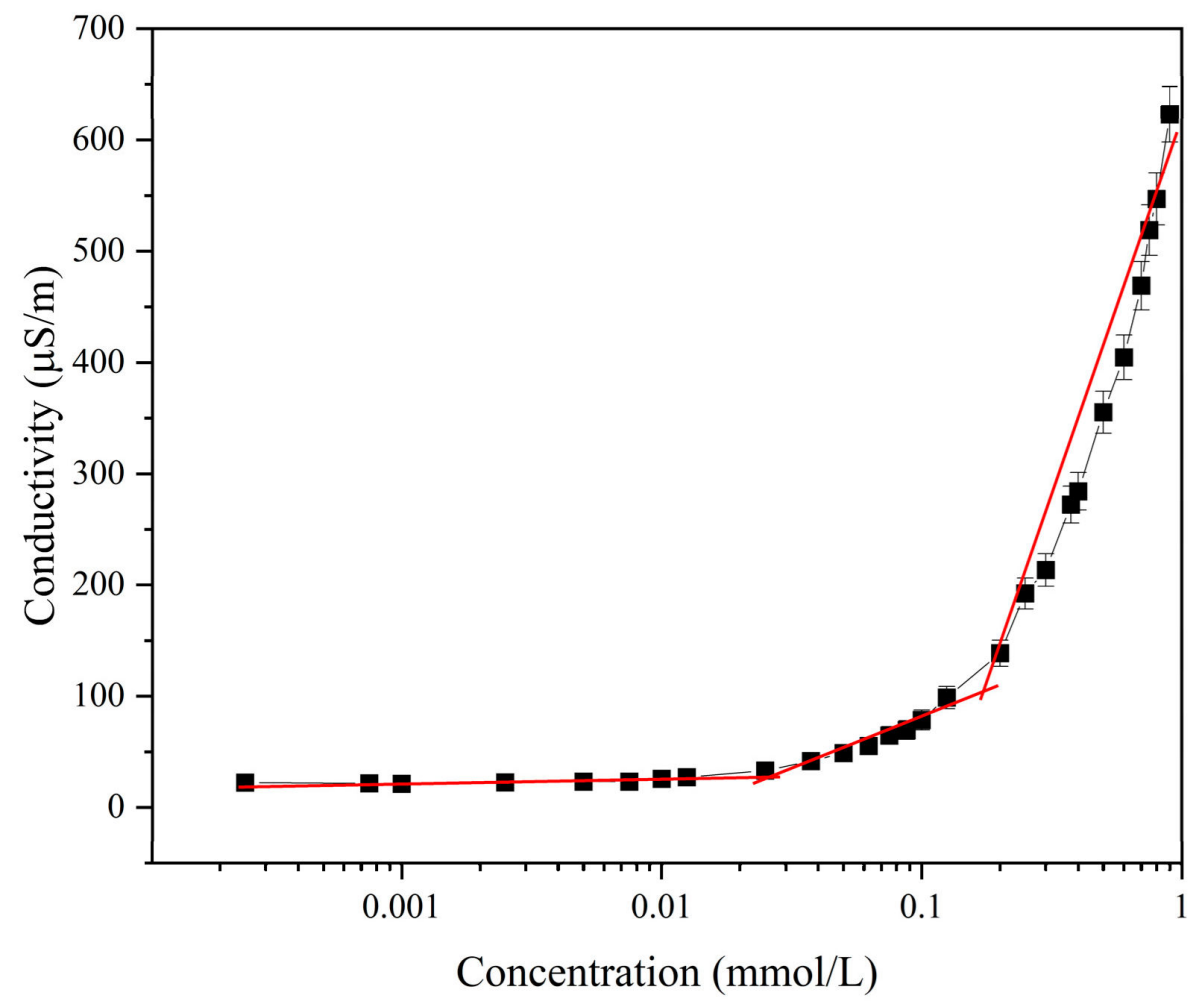
Variation in the electrical conductivity of the aqueous Tz-6C_12_QC solution with concentration at 25.0 °C, (Black is the conductivity curve, and red is the linear fitting line.)

A plot of the conductivity of the Tz-6C_12_QC solution as a function of concentration is shown in Figure 4. With increase in the Tz-6C_12_QC concentration, the conductivity of the solution increased significantly. However, the rate of conductivity increase was not exactly the same in the research range of the Tz-6C_12_QC concentration, and two distinct slope changes were observed in the plot of the conductivity of the Tz-6C_12_QC solution. The appearance of two inflection points can be attributed to the particularity of the structure and aggregation behavior of Tz-6C_12_QC. The critical aggregation concentration (CAC), which is the threshold above which compound aggregation occurs [43], and CMC, which is the concentration of micelle formation [41], obtained from the inflection points were 0.0194 and 0.172 mmol/L, respectively. The value of CAC indicated that the Tz-6C_12_QC molecules in solution formed aggregations (pre-micelles) when the concentration of Tz-6C_12_QC was higher than CAC and less than CMC. When the concentration exceeded CMC, these pre-micellar aggregations merged to form micelles. Typically, surfactant molecules undergo an association aggregation process to form micelles in solution as the concentration increases [20,42]. It was obvious that the CMC value calculated from the conductivity measurements was different from that obtained from the surface tension. The reason for the difference is that the test principle of the two methods is different. The principle of the surface tension measurement is based on the adsorption of surfactant molecules at the liquid/gas interface to reduce the interface tension. The CMC of the surface tension is determined by the inflection point of the surface tension curve, which means that the surfactant has reached the maximum adsorption at the liquid/gas interface. When the surfactant reaches the condition of maximum adsorption, it will start to form micelles in the bulk solution [41]. The measurement of CMC by conductivity relies on the fact that the presence of micelles in a solution can affect the ionic conductivity due to the differential mobility of the ions inside and outside the micelle. The principle of determining the CMC from conductivity testing lies in the fact that when there is a transition in the aggregation behavior of surfactants in a solution, there will be an inflection point on the conductivity curve. Due to the competitive behavior between adsorption at the surface and aggregation in the solution of surfactants, the maximum adsorption concentration of the surfactants at the surface and the aggregation concentration in solution are different, resulting in numerical differences between the CMCs determined by the surface tension and conductivity methods. Typically, surfactant aggregation occurs earlier than maximum adsorption at the interface [44]. Additionally, the six quaternary ammonium groups in the molecular structure of Tz-6C_12_QC tend to favor surfactant aggregation in solution over adsorption, resulting in a lower CMC value when determined by the conductivity compared to the surface tension. As the critical aggregation behavior has a relatively small impact on the surface tension of surfactant solutions, it is typically difficult to use this method to detect the surfactant CAC. The ionic surfactant CAC has usually been determined by conductivity measurement [43,45,46]. The conductivity test method provides more useful information about changes in the surfactant aggregation behavior. In order to better study the aggregation behavior of Tz-6C_12_QC, the CAC and CMC of the conductivity test were used as the basis for subsequent study.

Table 2 lists the distinct physicochemical properties of Tz-6C_12_QC and other traditional surfactants. The *Γ* and A of Tz-6C_12_QC were calculated from the average value curve of the surface tension by Equations (2) and (3). Compared with DTAB and 12-3-12-4-12-3-12, the surface excess concentration (Γ) of Tz-6C_12_QC was higher, indicating that the triazine rings as spacer groups could effectively overcome the charge repulsion between the hydrophilic head group and enhance the packing of Tz-6C_12_QC at the surface. Furthermore, the area occupied by each surfactant molecule of Tz-6C_12_QC was no larger than that of DTAB, suggesting that the triazine rings promoted the bending and entanglement of the hydrophobic chain in Tz-6C_12_QC. In contrast to the 12-3-12-4-12-3-12 alternate rigid spacer, the triazine ring spacer cannot induce bending and torsion. However, the Tz-6C_12_QC branch chain undergoes better entanglement, resulting in better interface aggregation ability compared to the former.

Generally, spacer groups can effectively improve the surface activity and the micellization propensity of surfactants. The hydrophilicity, hydrophobicity, size, and rigidity of spacers play an important role in the surface activity (Table 2). For the 1,3,5,7-tetrakis(dimethylhexylammonioacetoxy)-adamantane, tetrabromide (AD-6) surfactant with a rigid spacer-based alkane structure with low-polarity spacers, the introduction of low-polarity spacers increased the surface activity to a lesser extent compared to that for dimeric(1,2-bis(dodecyldimethylammonio)ethane dibromide (2RenQ) and 12-2-12 and Tz-6C_12_QC, 3C_4_NAc-Tz with polar spacers (moderate to high polarity). In addition, surfactants with a reasonable length of the hydrophobic group can exhibit enhanced surface activity. The introduction of a triazine spacer in Tz-6C_12_QC can increase its charge density and reduce the repulsion between the ionic head groups, while the tighter branched chain entanglement structure can reduce the space occupied by it. Therefore, Tz-6C_12_QC has better surface activity than traditional surfactants.

### 3.3. Thermodynamic Analysis of Micellization

The thermodynamics of micellization were investigated to determine the changes in the micellization behavior at different temperatures. The degree of counter-ion ionization (*α*) and the binding ability of the counter ion (*β*) of Tz-6C_12_QC were estimated by the conductivity method. The values of *α* and *β* can be calculated using Equations (18) and (19). $\Delta G_{m}^{0}$, $\Delta H_{m}^{0}$, and $\Delta S_{m}^{0}$ are the Gibbs free energy, enthalpy, and entropy of micellization, respectively. These thermodynamic parameters can be calculated using *X*_CMC_ and *β* based on Equations (20)–(22). The values of CMC, *α*, *β*, and the thermodynamic parameters are listed in Table 3.

(18) $$\alpha =S_{2}/S_{1}$$

(19) β=1−α

(20) $$\Delta G_{m}^{0}=RT\left(1+2\beta \right)\delta lnX_{CMC}/\delta T$$

(21) $$\Delta H_{m}^{0}=-RT\left(1+2\beta \right)\delta lnX_{CMC}/\delta T$$

(22) $$\Delta S_{m}^{0}=\left(\Delta H_{m}^{0}-\Delta G_{m}^{0}\right)/T$$

where *S*_1_ and *S*_2_ are the slopes of the linear conductivity versus concentration plots for concentrations below and above the CMC, respectively. *X_CMC_* is the molar fraction corresponding to the CMC, R is the gas constant, and T is the absolute temperature. CMC, α, β, and the thermodynamic parameters of Tz-6C_12_QC were calculated from the average value curve of conductivity by Equations (18)–(22) and are shown in Table 3.

With increase in temperature, the degree of counter-ion ionization of Tz-6C_12_QC increased, and the binding ability of the counter ion of Tz-6C_12_QC decreased and became negative. This phenomenon can be attributed to the existence of a certain number of antiparticles entering the solution from the inside of the micelles during the formation of micelles, which corresponds to the process of solvent molecules being expelled from the membrane during the formation of vesicles in the DPD simulation in Section 3.5. Similar phenomena were reported in Wang’s work [52]. Additionally, this is consistent with the rapid increase when the Tz-6C_12_QC concentration is greater than the CMC in conductivity. The negative value of $\Delta G_{m}^{0}$ of Tz-6C_12_QC (Table 3) indicates that micellization occurs spontaneously, while the negative value of $\Delta H_{m}^{0}$ indicates that micellization is an exothermic process. The positive value of $\Delta S_{m}^{0}$ suggests an increased degree of disorder in the system, thus facilitating the formation of micelles. This is because the “iceberg” region formed by water around the hydrophobic chain of Tz-6C_12_QC in an aqueous solution is destroyed [53]. Furthermore, the $\left|-T\Delta S_{m}^{0}\right|$ values are higher than the $\left|\Delta H_{m}^{0}\right|$ values for all systems, indicating that the micellization process is entropy-driven [54]. It is known that the hydrophobic effect leads to an increase in entropy upon micellization [55].

### 3.4. Shear Rheology Behavior

**Figure 5:**
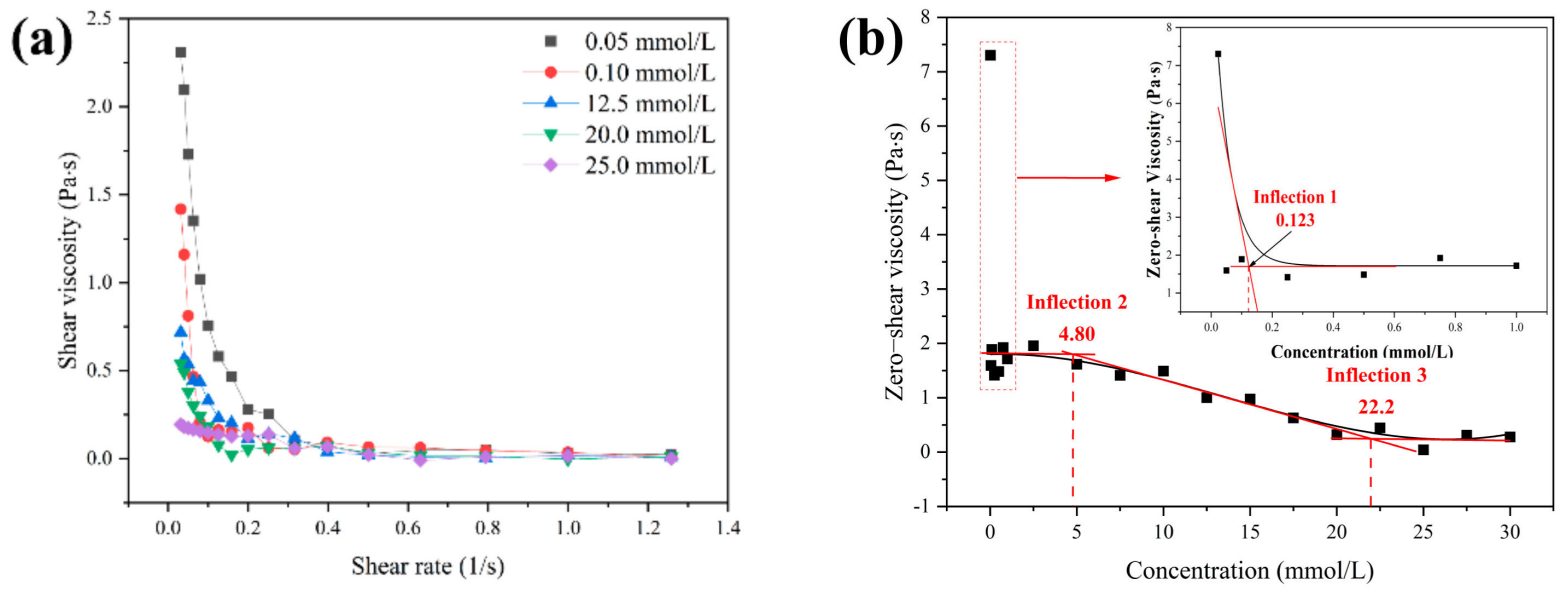
(**a**) Variation in the shear viscosity of the aqueous Tz-6C_12_QC solutions with concentration at 25.0 °C, (**b**) Variation in the zero-shear viscosity of the aqueous Tz-6C_12_QC solutions with concentration at 25.0 °C (Black is the shear-viscosity fitting curve, and red is the curve tangent.).

The shear rheology behavior of the Tz-6C_12_QC solutions was determined by rheology measurements. As shown in Figure 5a, the Tz-6C_12_QC solutions all showed a tendency for the shear viscosity to decrease with increasing shear rate. In addition, the initial shear viscosity of the solution gradually decreased as the Tz-6C_12_QC concentration increased. The zero-viscosity of the Tz-6C_12_QC solutions with different concentrations is shown in Figure 5b. With increase in the Tz-6C_12_QC concentration, the zero-shear viscosity of the solution showed an obvious decreasing trend. However, the rate of increase in the zero-shear viscosity was clearly different over the concentration range studied. With increase in the Tz-6C_12_QC concentration, the zero-shear viscosity of the solution decreased significantly, and when the concentration was greater than the first inflection point, called inflection 1, the decrease rate of the zero-shear viscosity of the solution became smaller. The value of inflection 1 was 0.123 mmol/L, which was close to the CMC measured by the conductivity. The phenomenon of the zero-shear viscosity reducing with increasing concentration indicated that the molecules aggregated in this concentration range [56]. As the concentration increased (greater than inflection 1 and less than inflection 2, which was 4.80 mmol/L), there was no significant change in the zero-shear viscosity of the Tz-6C_12_QC solution. When the concentration was higher than 0.123 mmol/L and less than 4.80 mmol/L, the Tz-6C_12_QC molecules formed stable micelles in solution. When the concentration was greater than inflection 2 and less than inflection 3, which was 22.2 mmol/L, the zero-shear viscosity of the Tz-6C_12_QC solution decreased with increasing concentration, indicating the presence of micelle transition behavior in this concentration range. Within this concentration range, as the concentration of Tz-6C_12_QC increased, the stable micelles formed in the previous stage began to aggregate and fuse. When the concentration was greater than inflection 3, the zero-shear viscosity of the solution showed no significant change. This indicated that new stable micelles were formed [56,57,58,59,60]. A schematic diagram of the above transformation process is shown in Figure S2.

**Figure 6:**
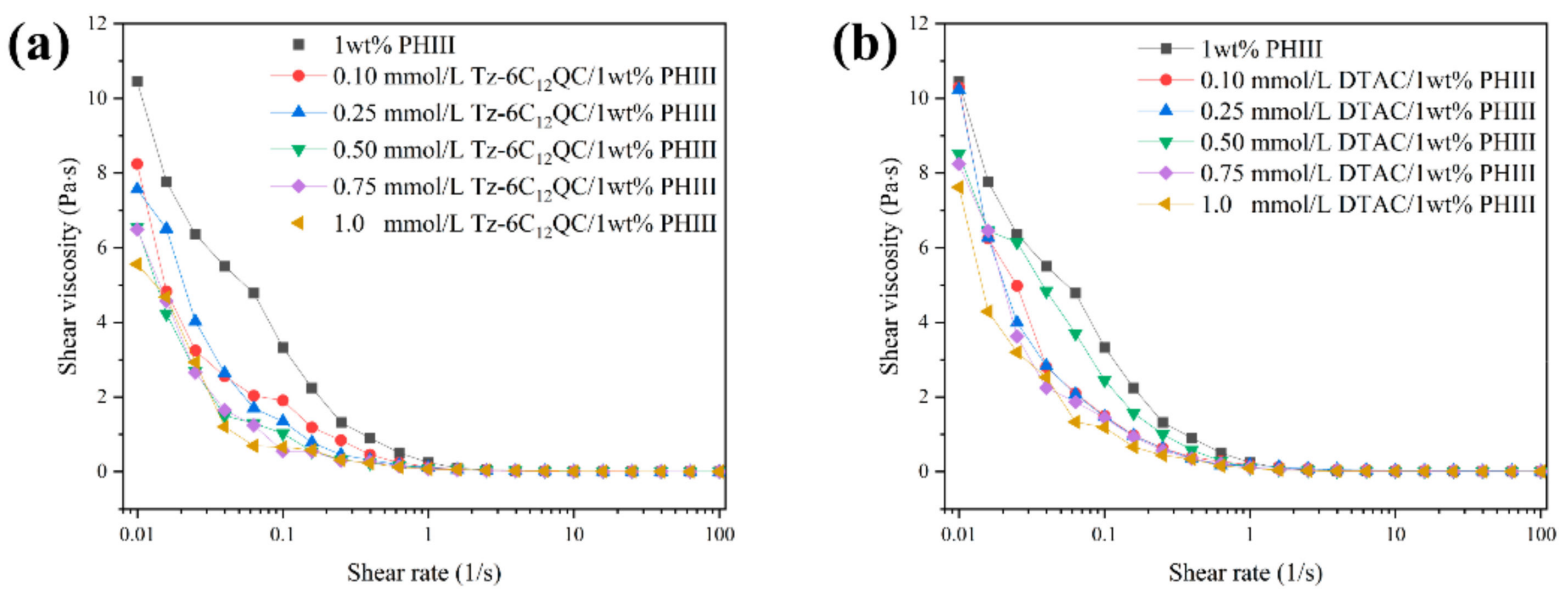

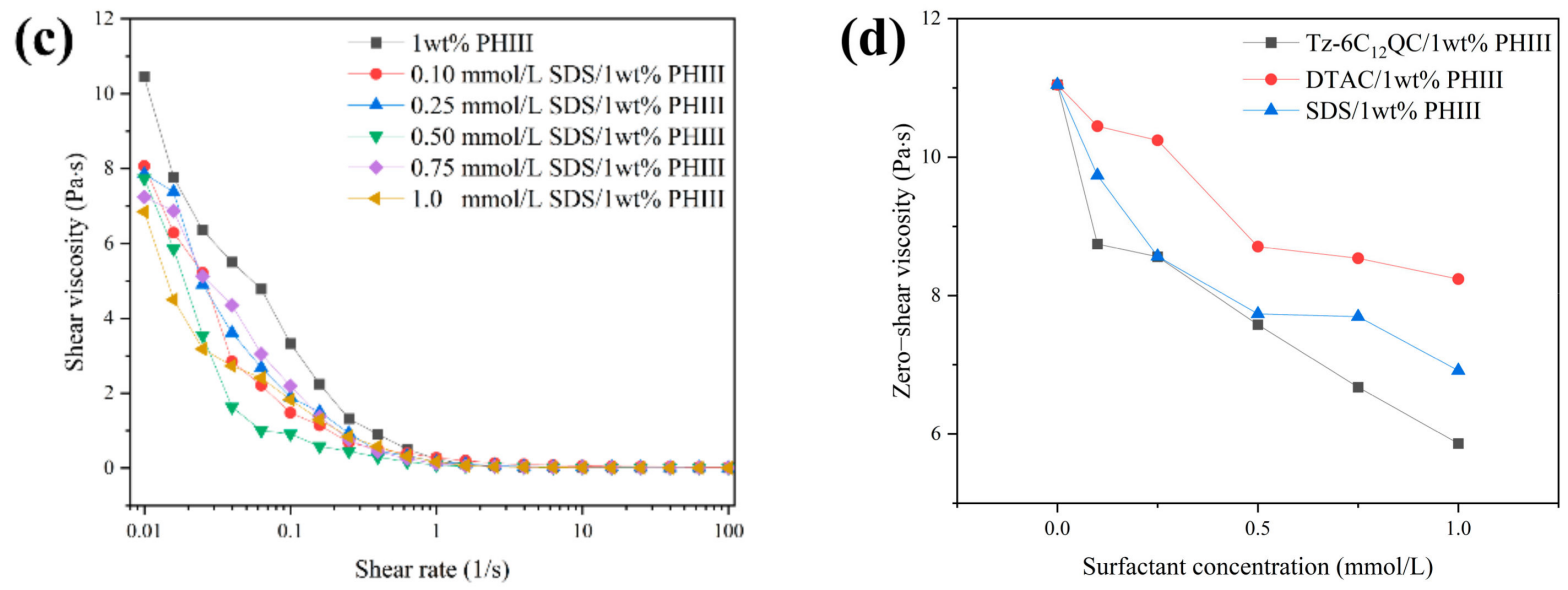
Variation in the shear viscosity of the aqueous surfactant/PHIII solutions with concentration at 25.0 °C, (**a**) Tz-6C_12_QC, (**b**) DTAC, (**c**) SDS. (**d**) Variation in the zero-shear viscosity of the aqueous surfactant/PHIII solutions with concentration at 25.0 °C.

The effect of surfactants on reducing the viscosity in a surfactant/polymer system is an important basis for their application in chemical-enhanced oil recovery (cEOR). The rheology of Tz-6C_12_QC/PHIII was characterized by shear rheology on the basis of the low viscosity of the Tz-6C_12_QC solutions (Figure 6). The rheology of Tz-6C_12_QC/PHIII was compared with traditional monomeric surfactants DTAC and SDS. In addition, DTAC and Tz-6C_12_QC have the same hydrophilic group and the same length of hydrophobic chain. SDS has the same length of hydrophobic chain and a different hydrophilic group to Tz-6C_12_QC. With increase in the Tz-6C_12_QC concentration, the initial shear viscosity of the solution decreased from 10.47 Pa∙s to 5.56 Pa∙s, which was lower than the 7.62 Pa∙s of the traditional monomer surfactant DTAC and 6.85 Pa∙s of SDS (Figure 6a–c). The zero-shear viscosity of the surfactants/PHIII with different concentrations is shown in Figure 6d. With increase in the surfactant concentration, the zero-shear viscosity of the solution showed an obvious decrease. With increase in the Tz-6C_12_QC concentration, the zero-shear viscosity of the solution decreased from 11.04 Pa∙s to 5.86 Pa∙s, which was lower than the 8.24 Pa∙s of the traditional monomer surfactant DTAC and 6.91 Pa∙s of SDS (Figure 6d). The reason is that the triazine spacer of Tz-6C_12_QC provides a better coating ability, which has a better coating and barrier effect on the PHIII molecules in solution, and reduces the hydrogen bonding between the PHIII molecules and destroys its physical cross-linking network [61]. Therefore, Tz-6C_12_QC has a greater viscosity-reducing effect.

### 3.5. Size, Distribution, and Morphology Analysis of Tz-6C_12_QC Aggregates

**Figure 7:**
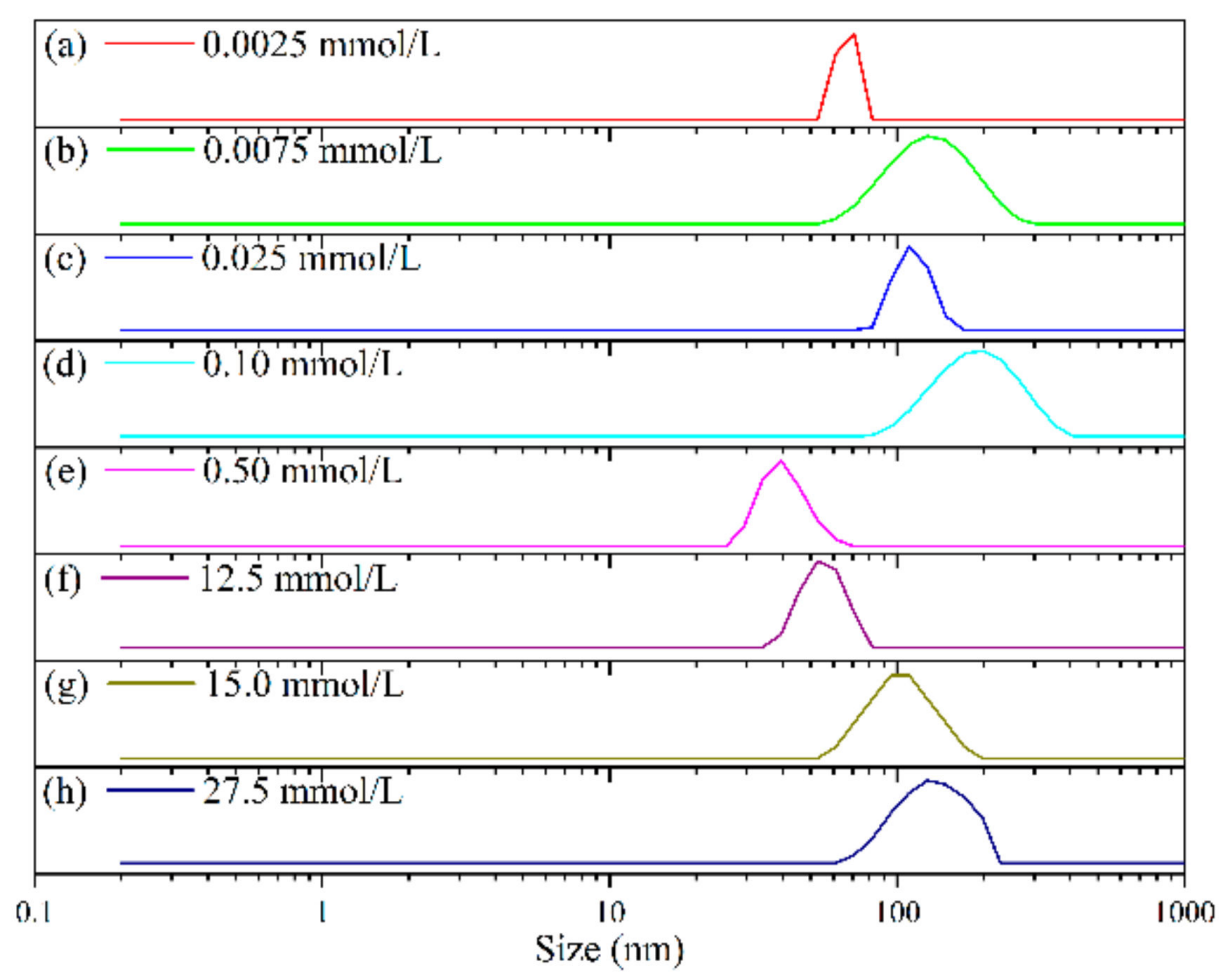
DLS curves of the size distribution of Tz-6C_12_QC at various concentrations.

**Figure 8:**
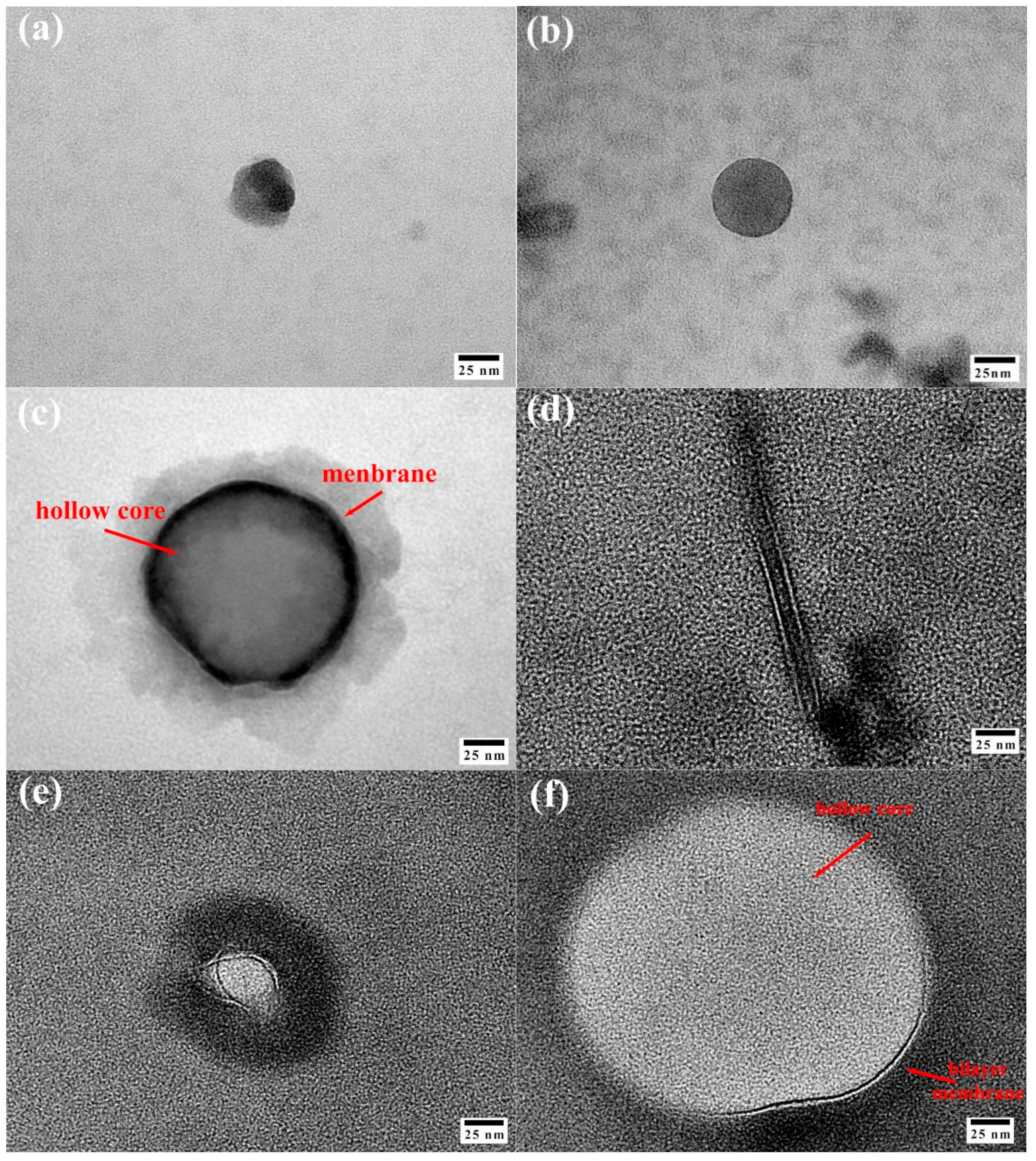
TEM micrographs of Tz-6C_12_QC aggregates formed at a concentration of (**a**) 0.00075 mmol/L, (**b**) 0.05 mmol/L, (**c**) 0.25 mmol/L, (**d**) 12.5 mmol/L, (**e**) 20.0 mmol/L, (**f**) 25.0 mmol/L.

**Figure 9:**
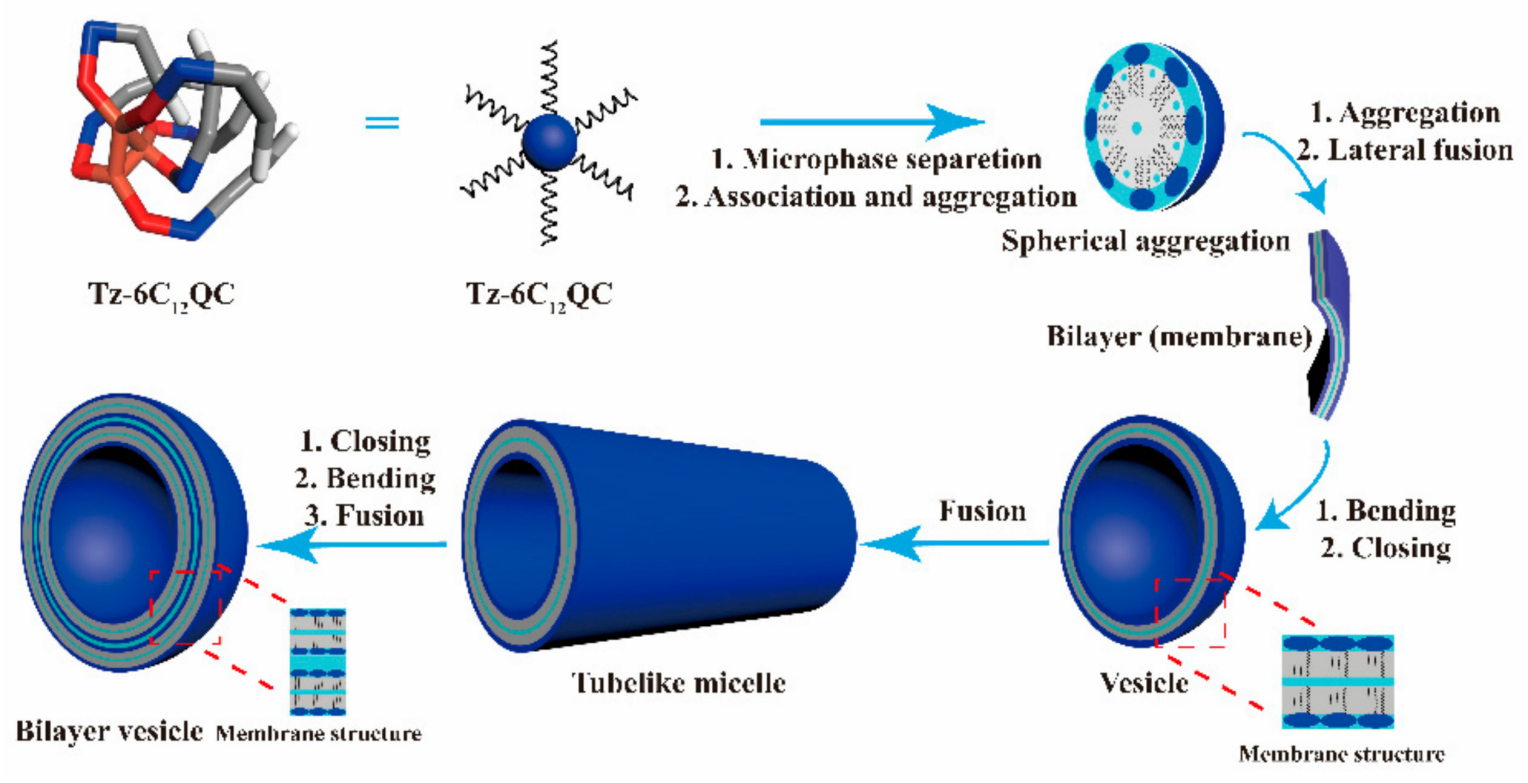
Schematic of the self-assembly behavior of Tz-6C_12_QC with increasing concentration.

DLS was employed to investigate the influence of the Tz-6C_12_QC concentration on the aggregate size and size distribution. With increasing Tz-6C_12_QC concentration, the Tz-6C_12_QC aggregates exhibited four distinguishable regions in terms of the size and size distribution (Figure 7). The aggregate morphologies at different concentrations were visualized by TEM (Figure 8). In the first region, where the Tz-6C_12_QC concentrations were much lower than the CAC, the Tz-6C_12_QC molecules formed an association in solution. The TEM results revealed associations of Tz-6C_12_QC molecules with a diameter of about 10 nm in Figure 8a, which is attributed to the association between monomeric molecules. These associations grew, and the distribution broadened with increasing concentration. The average particle size also increased from 66.69 to 135.53 nm (Figure 7a,b). In the second region, at concentrations above the CAC but below the CMC, the associations of Tz-6C_12_QC molecules fused together to form spherical aggregations (Figure 8b), which is similar to the findings in Vlachy‘s work [55]. In the third region, at concentrations above the inflection 2 point but below the inflection 3 point, derived from rheology measurements, the spherical aggregations fused to the vesicles (Figure 8c). The micelles had an obvious hollow structure and membrane structure. Moreover, the Tz-6C_12_QC molecules formed vesicles that did not show any significant change in size with further increase in concentration (Figure 7e,f). This was because more vesicles were formed by the Tz-6C_12_QC molecules with increasing concentration. When the Tz-6C_12_QC concentration was higher than inflection 3, the Tz-6C_12_QC molecules formed bilayer vesicles. During the fusion of vesicles to form tube-like micelles (Figure 8d), as reported in Akiyoshi’s works [62] and Ravoo’s works [63], tube-like micelles that underwent bending (Figure 8e) and fusion could form bilayer vesicles (Figure 8f). Additionally, during tube-like micelle formation in the process of development of the bilayer vesicles, the membrane structure was well-maintained. With increase in the Tz-6C_12_QC concentration, the bilayer vesicles fused and the micelle size increased. A schematic of the Tz-6C_12_QC self-assembly behavior is shown in Figure 9.

### 3.6. DPD Simulation of the Self-Assembly Behavior of Tz-6C_12_QC

**Figure 10:**
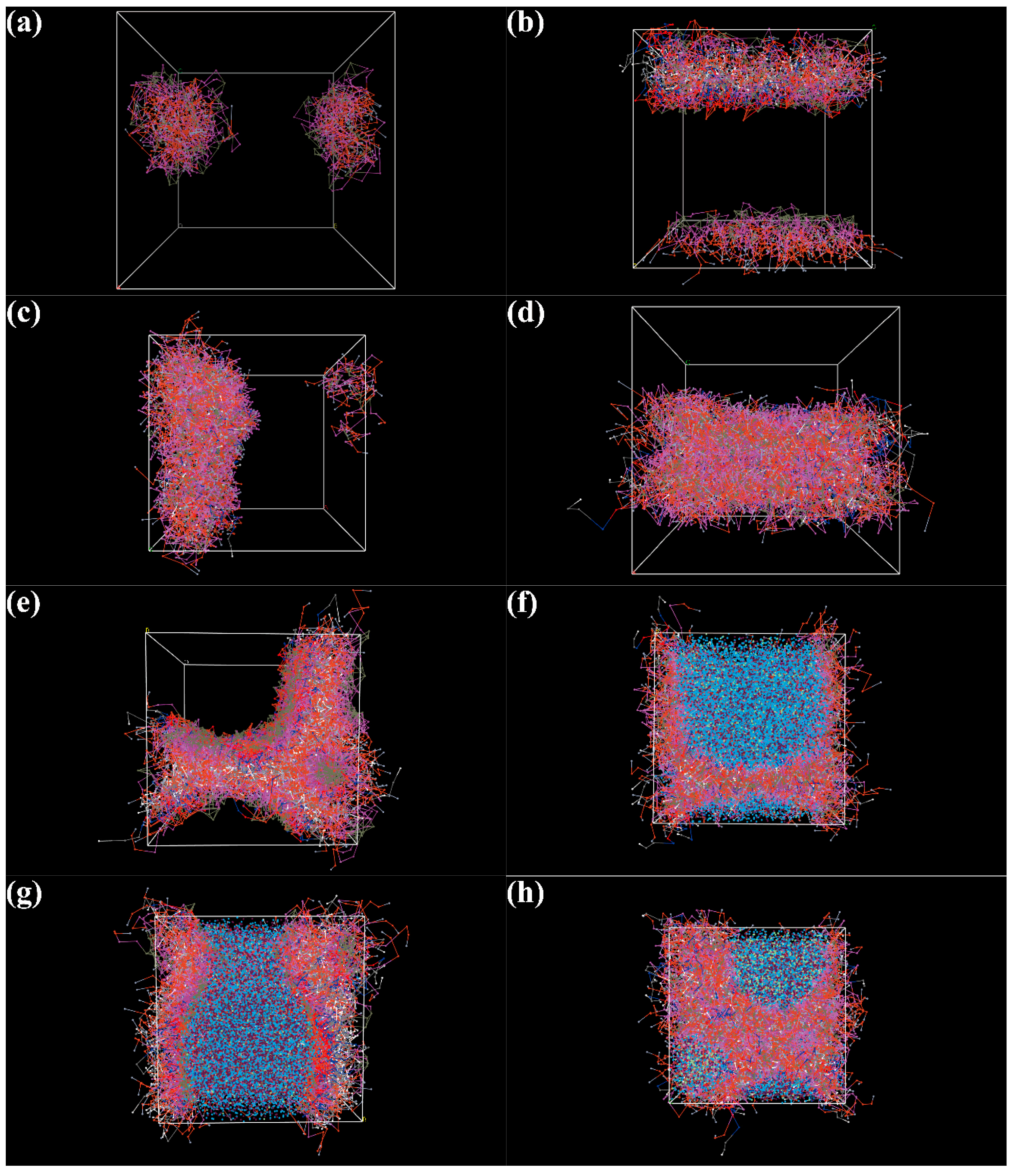
Micelles at different Tz-6C_12_QC concentrations: (**a**) 4.73%, (**b**) 11.0%, (**c**) 19.5%, (**d**) 32.0%, (**e**) 47.1%, (**f**) 55.9%, (**g**) 61.6%, and (**h**) 71.3%.

**Figure 11:**
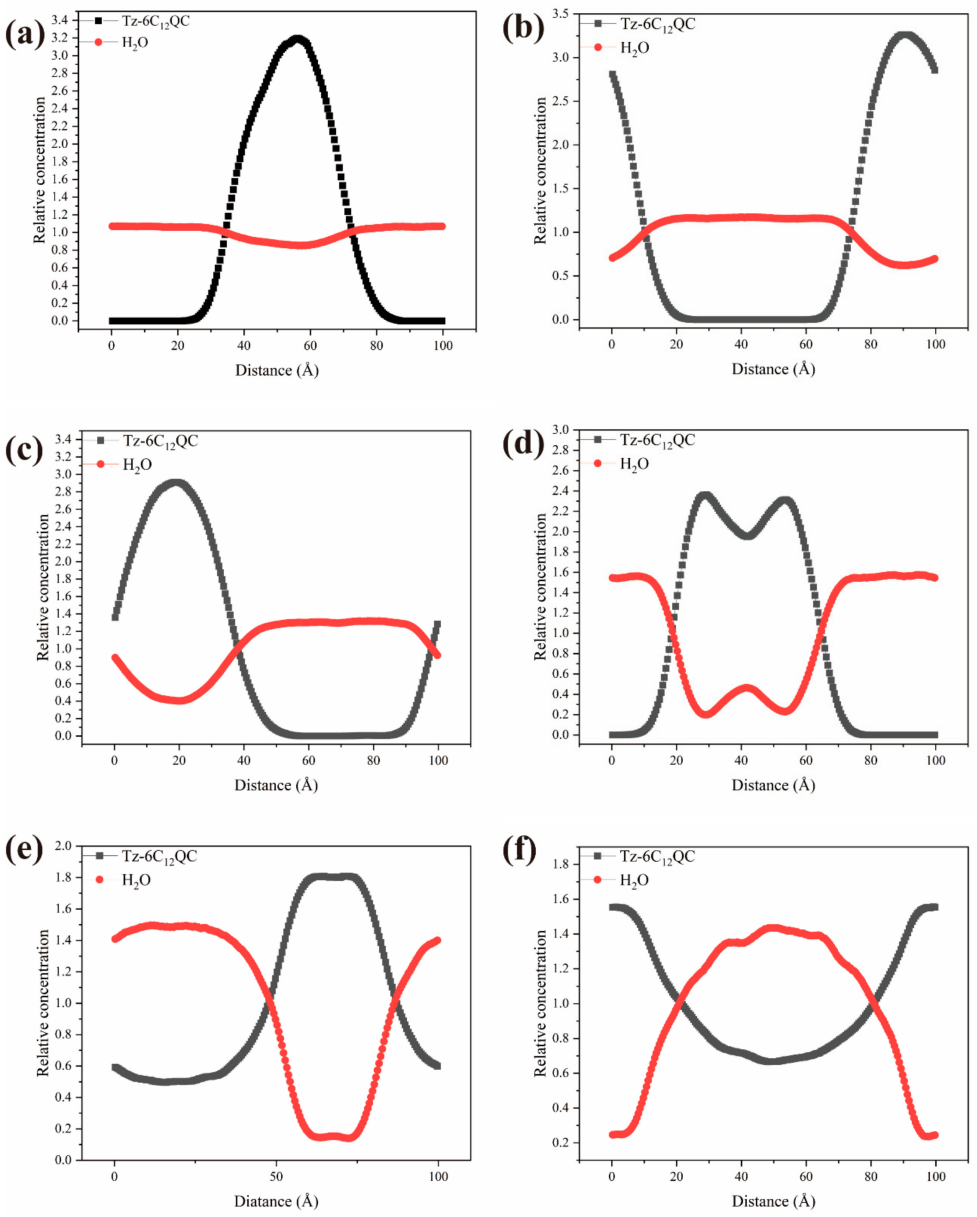

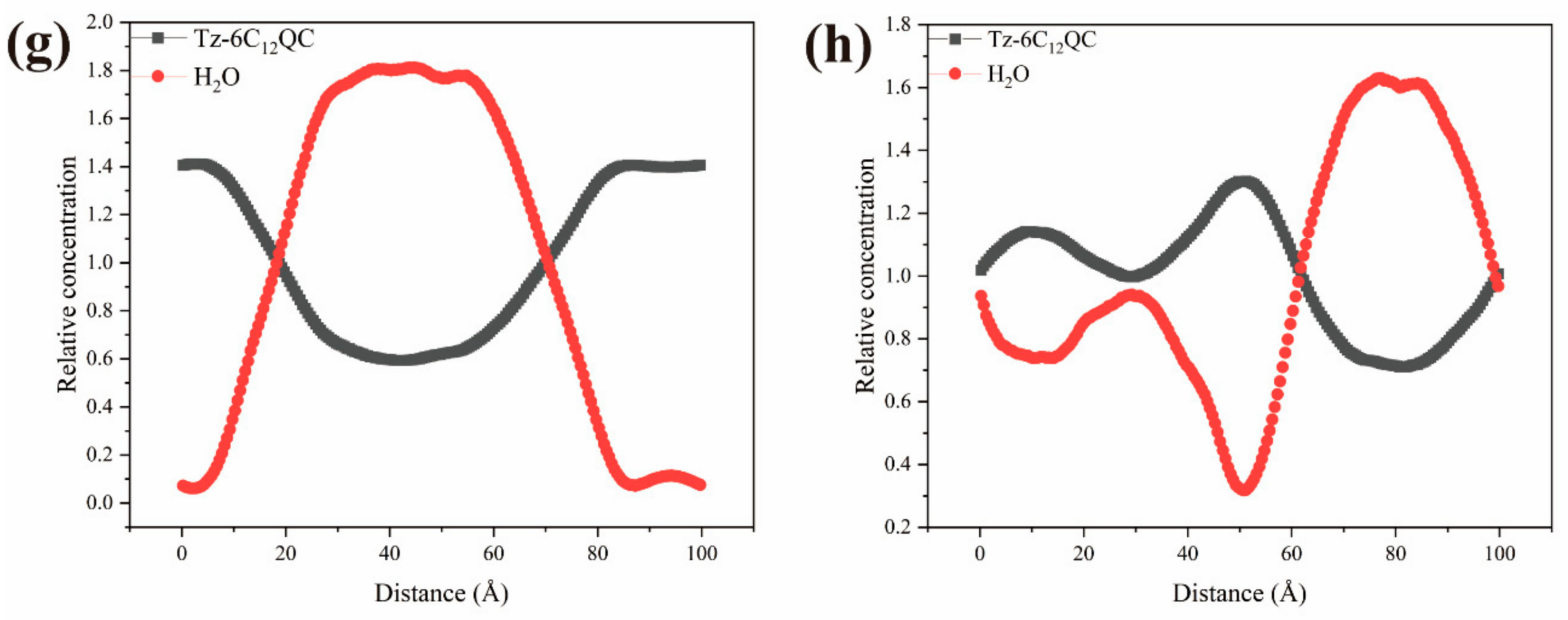
Relative concentration profiles for different numbers of Tz-6C_12_QC molecules: (**a**) 4.73%, (**b**) 11.0%, (**c**) 19.5%, (**d**) 32.0%, (**e**) 47.1%, (**f**) 55.9%, (**g**) 61.6%, and (**h**) 71.3%.

**Figure 12:**
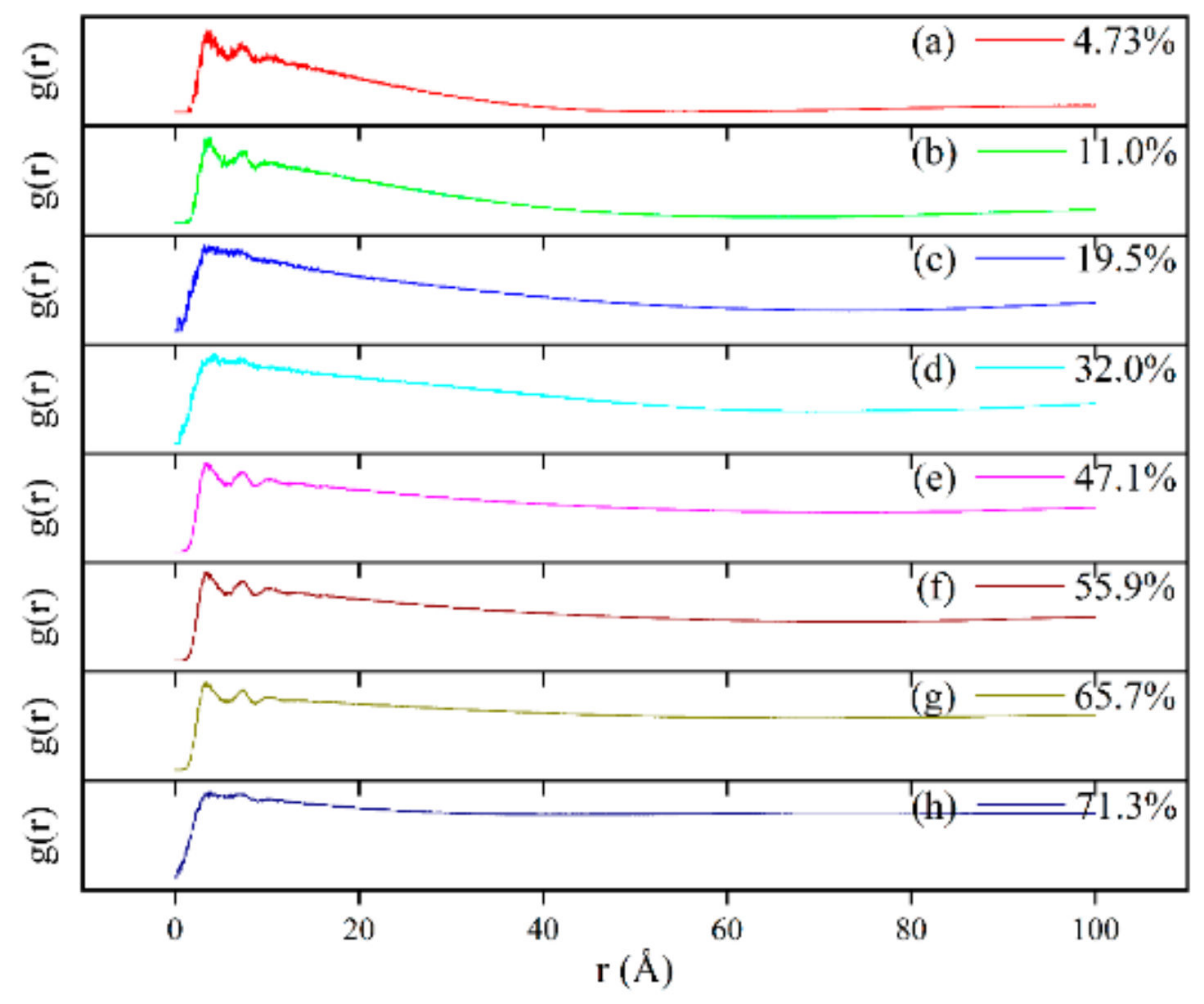
RDF curves of Tz-6C_12_QC: (**a**) 4.73%, (**b**) 11.0%, (**c**) 19.5%, (**d**) 32.0%, (**e**) 47.1%, (**f**) 55.9%, (**g**) 61.6%, and (**h**) 71.3%.

DPD simulations were performed to investigate the aggregation behavior of Tz-6C_12_QC at different concentrations. Due to the difficulty of expressing the molar concentration in DPD simulations, a commonly used number fraction is adopted to approximate the variation in the Tz-6C_12_QC content in the solution. With increasing concentration, Tz-6C_12_QC sequentially formed spherical micelles (Figure 10a), rod-like micelles (Figure 10b), bilayers (Figure 10d), vesicles (Figure 10f), tube-like micelles (Figure 10g), and bilayer vesicles (Figure 10h) in solution. The relative concentration profiles of Tz-6C_12_QC and water, indicating the relative distribution of the Tz-6C_12_QC and water molecules in the micelles, are shown in Figure 11. At low Tz-6C_12_QC concentrations in aqueous solutions, Tz-6C_12_QC molecules formed spherical micelles in which the water molecules were uniformly distributed (Figure 10a). As the Tz-6C_12_QC concentration increased, these spherical micelles fused together to form rod-like micelles (Figure 10b) that eventually formed bilayers (Figure 10d). At this point, two peaks were observed in the relative concentration profile of Tz-6C_12_QC (Figure 11d). In addition, during the formation of rod-like micelles from spherical micelles, water molecules inside the micelles were not expelled (Figure 11d). The radial distribution function (RDF, g(r)) of Tz-6C_12_QC shows the relative distance of the Tz-6C_12_QC molecules and can reveal changes in the micelle structure during the conversion of the aggregate morphology. Figure 12 shows the RDF for varying concentrations of Tz-6C_12_QC. There were no structural changes during the conversion from globular to rod-like micelles (Figure 12a,b). As shown in Figure 11b,d, the thickness of the micelles changed from 39.00 to 73.00 Å owing to the stretching of the hydrophobic chains, indicating that the thickness of the bilayers was slightly higher than the diameter of the rod-like micelles. During the formation of bilayers, a small quantity of water molecules was expelled because of the formation of hydrophobic layers between the two layers of micelles. The structure of the micelles changed and, consequently, a hump was observed in the RDF curve. However, a few water molecules were present in the gap between the two hydrophobic groups that constituted the hydrophobic layer (Figure 11c,d). Due to insufficient structural strength to sustain infinite growth of the micelle size, the bilayers bent and fused together to form vesicles with increasing Tz-6C_12_QC concentration (Figure 10f). During this process, the thickness of the bilayers decreased (Figure 12e,f). The layered structure changed due to bending of the bilayers (Figure 11e,f). With further increase in the Tz-6C_12_QC concentration, the vesicles fused together to form tube-like micelles (Figure 10g) that finally formed bilayer vesicles (Figure 11h). The relative concentration profile suggested the existence of two membrane layer structures (Figure 11h). The relative concentration profiles in Figure 11f–h and the RDF in Figure 12f–h indicate that the membrane structure did not undergo any significant change with the formation of vesicles, tube-like micelles, and bilayer vesicles.

## 4. Conclusions

A hexameric cationic quaternary ammonium salt surfactant Tz-6C_12_QC was synthesized. Surface tension and conductivity measurements undertaken revealed better surface activity of Tz-6C_12_QC than that of traditional surfactants and that Tz-6C_12_QC micellization was a spontaneous, exothermic, and entropy-driven process. Rheological measurements revealed that Tz-6C_12_QC had a better viscosity reduction ability for polyacrylamide solutions than conventional surfactants. With increasing concentration, Tz-6C_12_QC molecules gradually formed Tz-6C_12_QC molecules associations, spherical aggregations, vesicles, tubular micelles, and bilayer vesicles in solution as shown by DLS and TEM. DPD simulations showed the process of formation of bilayers and the process by which bilayers bend and fuse to from vesicles. The vesicles, tubular micelles, and bilayer vesicles observed in the simulation studies confirmed the experimental results, indicating the rationality of employing DPD simulations for studying the self-assembly behavior of polysurfactants with rigid spacers. In addition, PHIII/surfactant rheological measurements indicated the potential of Tz-6C_12_QC in viscosity-reducing applications. This study on hexameric quaternary ammonium salt surfactants, using a combined experimental and computational approach, not only suggests a new synthetic route for highly active surfactants, but also provides a valuable reference for exploring the aggregation behavior of polysurfactants.

## Figures and Tables

**Table 1:** Relevant parameters of DPD simulation at 298 K.

Bead Type	Fragment	Repulsive Parameters *a_ij_*						
		CN_2_	CO(CH_2_)_2_	N^+^(CH_3_)_2_	(CH_2_)_4_	(CH_2_)_3_CH_3_	Cl^−^	(H_2_O)_4_
A	CN_2_	105.00						
B	CO(CH_2_)_2_	106.89	105.00					
C	N^+^(CH_3_)_2_	107.33	105.30	105.00				
D	(CH_2_)_4_	109.11	105.38	105.28	105.00			
E	(CH_2_)_3_CH_3_	109.05	105.48	105.34	105.01	105.00		
F	Cl^−^	106.89	106.58	107.18	108.86	109.05	105.00	
G	(H_2_O)_4_	104.82	105.38	105.36	106.81	107.12	103.47	105.00

**Table 2:** Physicochemical properties of Tz-6C_12_QC and other surfactants.

Surfactant	CMC [mmol/L]	γ_max_ [mN/m]	10^6^Γ [mol/m^2^]	A [nm^2^/Molecule]
DTAB [42]	14	38.6, 38.9	3.42	0.49
2RenQ [47]	0.90	21.4	2.31	0.72
12-2-12 [48]	0.97	32.4	-	0.1
3C_4_NAc-Tz [49]	0.19	32.9	3.54	0.47
12-3-12-4-12-3-12 [50]	0.06	-	1.30	0.64
AD-6 [51]	6	-	-	-
Tz-6C_12_QC	0.34	28.27	4.67	0.356

CMC in this table was determined by the surface tension method, and the structure of the surfactants in this table was provided in Figure S1.

**Table 3:** CMC, α, β, and thermodynamic parameters of Tz-6C_12_QC at different temperatures.

T [°C]	α	β	CMC [mmol/L]	$\Delta G_{m}^{0}$ [J/mol]	$\Delta H_{m}^{0}$ [J/mol]	$\Delta S_{m}^{0}$ [J/mol∙K]	$-T\Delta S_{m}^{0}$ [J/mol]
25	0.997	0.003	0.172	−31,623.19	−252.79	105.22	−31,370.40
50	1.06	−0.06	0.173	−30,201.48	−259.76	92.66	−29,941.71
75	1.11	−0.11	0.175	−29,042.51	−267.25	82.65	−28,775.26

CMC in this table was determined by the conductivity method.

## Data Availability

All relevant data are within the manuscript and Supplementary Materials.

## Supplementary Materials

The following supporting information can be downloaded at: https://www.mdpi.com/article/10.3390/polym15224396/s1, Figure S1: The structure of surfactants in Table 2; Figure S2: The schematic diagram of the relationship between zero-viscosity changes and micellar behavior.

## Abbreviations

DPD: dissipative particle dynamics; CMC, critical micelle concentration; CAC, critical aggregation concentration; FT-IR, Fourier-transform infrared spectroscopy; NMR, nuclear magnetic resonance spectroscopy; DLS, dynamic light scattering; TEM, transmission electron microscopy; RDF, radical distribution function.
