# Supplementary material for: Synthesis and Aggregation Behavior of Hexameric Quaternary Ammonium Salt Surfactant Tz-6C12QC
Source: Polymers (Basel). 2023 Nov 13;15(22):4396. doi: 10.3390/polym15224396 (PMC10674742; doi:10.3390/polym15224396)
Supplement: Supplementary file 1 [file polymers-15-04396-s001.zip › polymers-2603997-supplementary.pdf]

polymers

MDPI

Supporting Materials: Synthesis and aggregation behavior of hexameric quaternary ammonium salt surfactant Tz-6C12QC

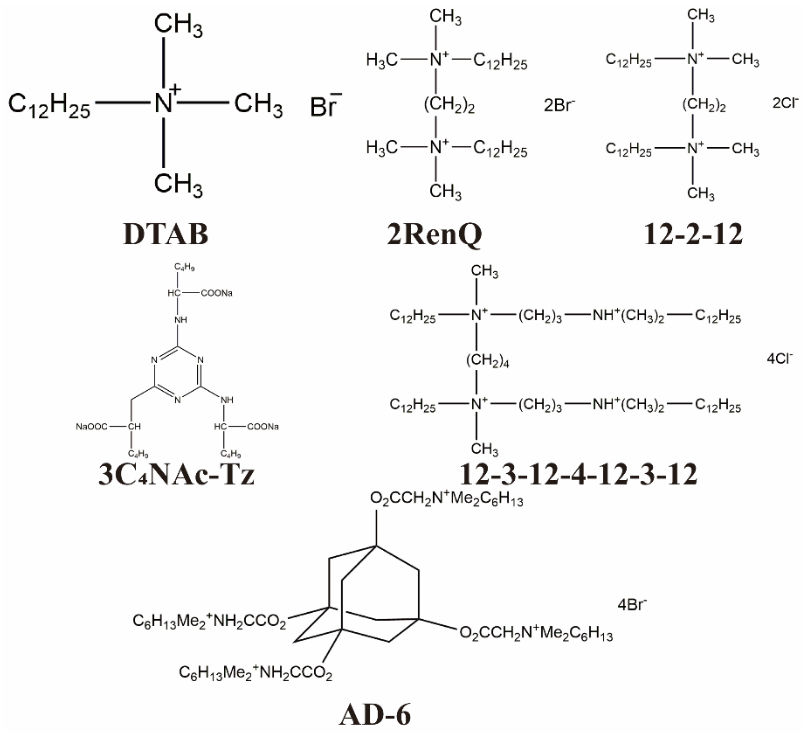

Figure S1. The structure of surfactants in Table 2.

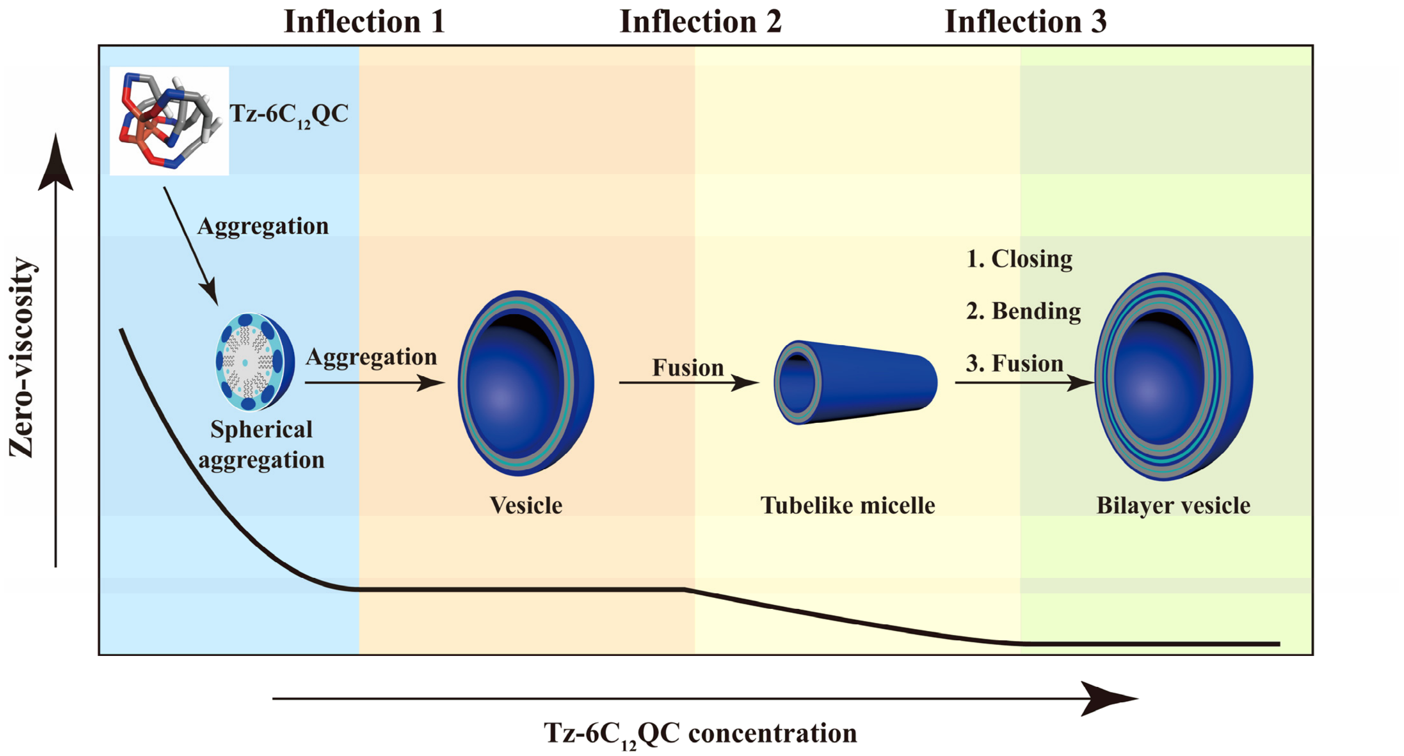

Figure S2. The schematic diagram of the relationship between zero-shear viscosity changes and micellar behavior.
